# Degeneration of the Injured Cervical Cord Is Associated with Remote Changes in Corticospinal Tract Integrity and Upper Limb Impairment

**DOI:** 10.1371/journal.pone.0051729

**Published:** 2012-12-12

**Authors:** Patrick Freund, Torben Schneider, Zoltan Nagy, Chloe Hutton, Nikolaus Weiskopf, Karl Friston, Claudia A. Wheeler-Kingshott, Alan J. Thompson

**Affiliations:** 1 Department of Brain Repair and Rehabilitation, University College London Institute of Neurology, University College London, London, United Kingdom; 2 Wellcome Trust Centre for Neuroimaging, University College London Institute of Neurology, University College London, London, United Kingdom; 3 Spinal Cord Injury Centre, Royal National Orthopaedic Hospital, University College London, London, United Kingdom; 4 Swiss Paraplegic Research, Nottwil, Switzerland; 5 NMR Research Unit, Department of Neuroinflammation, University College London Institute of Neurology, University College London, London, United Kingdom; Beijing Institute of Radiation Medicine, China

## Abstract

**Background:**

Traumatic spinal cord injury (SCI) leads to disruption of axons and macroscopic tissue loss. Using diffusion tensor imaging (DTI), we assessed degeneration of the corticospinal tract (CST) in the cervical cord above a traumatic lesion and explored its relationship with cervical atrophy, remote axonal changes within the cranial CST and upper limb function.

**Methods:**

Nine cervical injured volunteers with bilateral motor and sensory impairment and ten controls were studied. DTI of the cervical cord and brain provided measurements of fractional anisotropy (FA), while anatomical MRI assessed cross-sectional spinal cord area (i.e. cord atrophy). Spinal and central regions of interest (ROI) included the bilateral CST in the cervical cord and brain. Regression analysis identified correlations between spinal FA and cranial FA in the CST and disability.

**Results:**

In individuals with SCI, FA was significantly lower in both CSTs throughout the cervical cord and brain when compared with controls (p≤0.05). Reduced FA of the cervical cord in patients with SCI was associated with smaller cord area (p = 0.002) and a lower FA of the cranial CST at the internal capsule level (p = 0.001). Lower FA in the cervical CST also correlated with impaired upper limb function, independent of cord area (p = 0.03).

**Conclusion:**

Axonal degeneration of the CST in the atrophic cervical cord, proximal to the site of injury, parallels cranial CST degeneration and is associated with disability. This DTI protocol can be used in longitudinal assessment of microstructural changes immediately following injury and may be utilised to predict progression and monitor interventions aimed at promoting spinal cord repair.

## Introduction

Trauma to the spinal cord leads to retrograde and anterograde degenerative changes of central pathways [1]–[3]. Thus, axonal information flow is impeded and motor neurons below the site of injury are often deprived of supraspinal input [4]. This persistent lack of descending input may hinder clinical recovery. Recently, we and others showed that, following injury, the axonal and myelin integrity of the cranial corticospinal tract (CST) are reduced in specific motor areas [1], [2], [5] and correlate directly with spinal atrophy and cortical motor reorganisation. Moreover, the extent of spinal atrophy relates directly to disability [1], [6]. To explore these findings further we need to address the following questions: (1) How do the intrinsic changes within the atrophic spinal cord relate to central axonal changes in the cranial CST; (2) Does that degree of intrinsic spinal changes relate to manual dexterity independently of spinal atrophy [1].

Trauma induced microstructural tissue changes, although not visible on conventional MRI scans, alters free water diffusion [7] and this can be quantified by diffusion tensor imaging (DTI) [8]. In particular, fractional anisotropy (FA) has been reported as a marker of both axonal count [9] and myelin content [10]. We used this method in a cohort of SCI patients in the cervical cord as well as in the brain to investigate the degree of trauma-related abnormalities within cranial and spinal parts of the CST. DTI of the brain was thoroughly investigated in a previous report [5]. To answer our questions about the anatomical and behavioural correlates of white matter integrity at the spinal level, we use regression analysis to investigate the relationships between (1) the cross-sectional spinal cord area (i.e. spinal atrophy) measured with anatomical MRI, (2) changes in FA in the cervical cord and cranial CST measured by DTI, (3) clinical assessment of upper limb function.

## Subjects and Methods

### Subjects

From the same SCI patient cohort as previously reported [1], [5], [11], we studied ten male subjects, (level of lesion C5 to C8, mean age 45.7 years, post injury 14.9 years) who had bilateral upper and lower limb impairment (mean ASIA motor score 21.95). SCI subjects had no head or brain lesion associated with the trauma, or any history of seizure, medical or mental illness. All participants were free of MRI contraindications.

We also recruited ten gender matched right-handed healthy subjects within the same age range (mean age = 38.8 yrs, SD = 15.5, range = 25−65; p = n.s.) without any history of neurological or psychiatric illness.

Prior to the study, all participants gave informed, written consent. The study was approved by the Joint Ethics Committee of the Institute of Neurology at University College London and the National Hospital of Neurology and Neurosurgery, UK (ref: 08/0243).

### Clinical Assessment

All participants were assessed clinically using the 9-Hole Peg Test (9HPT) bilaterally [12]. Their maximum voluntary contraction (MVC) and performance on the Arm Action Research Test (ARAT) were tested with their dominant hand. The reciprocal of the average of two trials – for each hand of the 9HPT – and the average of two trials of the MVC were recorded. In three SCI subjects, the 9HPT was scored with the maximum time allowed for the 9HPT (300 sec) [13] as they were unable to perform with their non-dominant hand. To assess differences in motor performance between SCI subjects and controls, a two-sample t-test was used. A p-value<0.05 was considered significant.

### Image Acquisition

#### T1-weighted scan of brain and spinal cord

An optimized 3D-MDEFT sequence was used to obtain T1-weighted (T1w) structural images of the whole brain, brainstem and cervical cord (down to C5) [14] on a 1.5 T whole-body Magnetom Sonata MRI scanner (Siemens Medical Systems, Erlangen, Germany). The scan parameters were: isotropic 1 mm^3^ resolution, FoV 256×256 mm^2^, matrix 256×256, 176 sagittal partitions, repetition time = 12.24 ms, echo time = 3.56 ms, inversion time = 530 ms, flip angle 23°, fat saturation, bandwidth 106 Hz/Pixel. The acquisition time was 13 min 43 sec.

#### Diffusion tensor imaging (DTI) of the cervical cord

DTI was performed using a single shot echo planar imaging (EPI) sequence using the twice refocused spin-echo method for diffusion encoding [15]. The cervical cord data set consisted of 68 images: 7 images with a low b-value of 100 s*mm^–2^ and 61 directions with a high b-value of 1000 s*mm^–2^. The diffusion gradient directions in both subsets were uniformly distributed over the sphere according to [16]. The image volumes consisted of 20 axial slices with thickness of 5 mm and an in–plane resolution of 1.5×1.5 mm^2^, with no inter-slice gaps, acquisition matrix of 96×96, field of view of 144×144 mm^2^, and bandwidth 1408 Hz/Pixel. The acquisition was triggered from the peripheral pulse trace for reducing artefacts associated with cardiac induced motion [17]. The echo time was 90 ms and the repetition time was 180 ms. Interleaved slice acquisition order was chosen to avoid cross talk between adjacent slices. Two acquisitions with opposite read-out gradient polarity were acquired to correct for EPI induced geometric distortions [18]. The total scan time was approximately 20 minutes, depending on heart rate.

The two datasets obtained in the cervical cord with opposite phase encoding directions were combined into a single dataset with reduced geometric distortions [18]. Spinal volumes in image space were then sinc interpolated from a 96×96 acquisition matrix to a 192×192 image matrix, resulting in an in-plane resolution of 0.75 mm^2^. A diffusion tensor model was fitted to the interpolated data on a voxel-by-voxel basis using the open-source Camino software package (www.camino.org) (Cook et al., 2006). Before further processing, all images were checked for artefacts.

#### Diffusion tensor imaging (DTI) of the brain

To investigate the relationship between microstructural changes along the entire course of the CST (i.e. spinal cord and brain) we also acquired a brain DTI data set using a single shot echo planar imaging sequence as above [15]. Each data set consisted of 61 images with a b-value = 1000 s*mm^–2^ and 7 images with a b-value = 100 s*mm^–2^. Details of this sequence have been reported previously [19] and are therefore reported here only in brief: 2.3 mm isotropic resolution, FoV = 220×220 mm^2^, matrix size = 96×96, 60 axial slices, no inter-slice gaps, interleaved slice acquisition order, slice-to-slice repetition time = 160 ms, echo time = 90 ms, flip angle = 90°, readout bandwidth = 2003 Hz/Pixel, two repetitions, with inverted read-out polarity as described for the spinal cord DTI acquisition. The total acquisition time was 19 minutes. On the combined distortion-corrected data set [18], the diffusion tensor model was fit at each voxel using the RESTORE method [20] as implemented in Camino.

### Preprocessing for Voxel-based Quantification of DTI Data

Preprocessing steps from the VBM-DTI data set of the brain were described in detail previously [19] and are reported here in brief: i) *Segmentation*: a unified segmentation procedure [21] was used for bias correction and segmentation of the T1w image into GM, WM and cerebrospinal fluid (CSF). For each subject, this resulted in three images in T1w native image space, in which each voxel was assigned a probability of being GM, WM and CSF, respectively. ii) *Coregistration of FA and other DTI maps to WM*: For each subject the FA map was linearly coregistered to the corresponding WM probability map using 12 degrees of freedom and trilinear interpolation. This step exploited the similarity between the information in the WM probability map and the distortion corrected FA map to ensure that subject DTI data was in alignment with the corresponding T1w data. iii) *Creation of a WM mask in DTI space*: The WM probability map was resliced into DTI space using the inverse transformation from the previous step and binarized by thresholding at the same probability value>0.15 for consistency with the cervical cord DTI analysis. iv) *Application of WM mask to DTI maps*: The WM mask was applied to the registered FA map to create a subject-specific white-matter masked map of FA. v) *Non-linear registration of T1w image to MNI space*: A diffeomorphic non-linear image registration tool (DARTEL, [22]), was used to estimate the deformation fields required to warp the GM and WM probability maps from each subject T1w native image space into MNI space. vi) *Transformation of FA maps into MNI space*: The DARTEL deformation fields were applied to the corresponding FA maps and the white matter mask and smoothed by a 10 mm FWHM Gaussian kernel to account for inter-individual anatomical variability. vii) *Tissue specific smoothing compensation*: The smoothed warped FA map was divided by the smoothed warped white matter mask to compensate for the reduction of FA caused by smoothing [23], [24].

### Image Analysis

We examined i) cross-sectional spinal cord area and FA differences in both spinal and cranial ROIs between SCI patients and controls; ii) the correlation between FA of the whole cervical cord and cervical atrophy; iii) the correlation between spinal FA and clinical outcomes and iv) the correlation between spinal FA and cranial FA changes of the CST.

### Cross-sectional Spinal Cord Area Measurement

Previously, we reported the group difference of the *cross-sectional spinal cord area* using a standard semi-automated segmentation method on optimized 3D T1w scans [1], [14] in a study cohort including the subset of the current participants (see [19]).

### Region of Interests in the Cervical Cord – DTI Data

**Figure 1:**
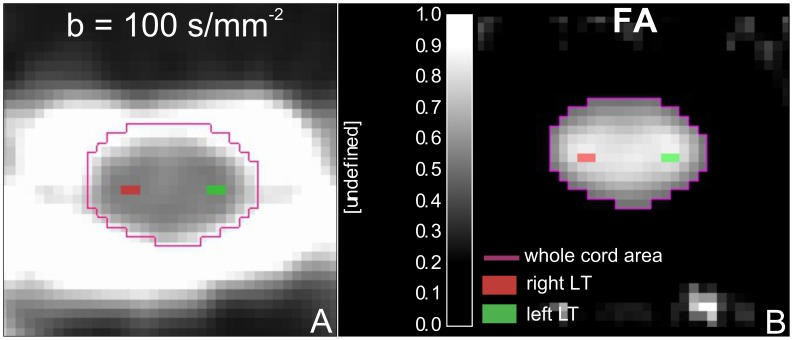
Axial FA image of the cervical cord (C1–C3) showing the locations of the two ROIs superimposed on the anatomoical location of the left and right corticospinal tracts. Note that ROIs were first drawn on the low diffusion weighted images (A) and then overlaid onto the diffusion maps (B).

To assess regional differences in FA, in each participant two ROIs were manually drawn on the average low-b-value image in native space between C1 and C3 covering the left and right CST running in the lateral columns (Fig. 1) similar to those ROIs used in [25]. To obtain the intra-observer coefficient-of-variation (CoV) for the FA of the right and left CST, a second experiment was run by the same observer (PF), who was blind to the results of the first experiment, 1 week later, on five randomly chosen participants. The mean CoV of the left and right CST was below 5%. As the FA of both CST sides in SCI patients and controls did not differ significantly the mean FA was calculated and used in the analysis regarding the associations between cervical FA and cranial *FA.* In addition, a ROI covering the whole spinal cord was determined using an automatic region growing segmentation algorithm [26] applied to the FA map with a lower threshold of FA ≤0.15 as the stopping criterion. We then calculated the mean FA value within each ROIs of the spinal cord. Based on the cross-sectional spinal cord area segmentation, whole cord FA measures were corrected for partial volume effects as described in [27].

### Regions of Interest (ROI) in the Brain – DTI Data

As we focused our investigations on degenerative axonal changes in the cranial CST we limited our search volume using a binary mask representing the left and right CST from the JHU white-matter tractography atlas [28]. The following bilateral ROIs containing the CST from the ICBM-DTI-81 white-matter labels atlas [29] were extracted and coregistered to the MNI template: pyramids, cerebral peduncle and posterior limb of the internal capsule.

### Statistical Analysis of Imaging Data

The spinal cord DTI data set of one patient was affected by motion artefacts and another patient had an incomplete DTI brain data set. Therefore the final analysis comprised nine SCI patients and ten controls for the group comparison of spinal FA and eight SCI patients and ten controls for the interaction between spinal FA and cranial FA.

### Cervical Cord DTI Data

We applied a two-sample t-test to investigate differences in FA of the left and right CST ROIs and the whole cervical cord ROI between SCI subjects and controls. Two multiple linear regression models were used to assess relationships between: (i) changes in the microstructure (using FA of the whole cervical cord ROI) and macrostructural changes (e.g. cord area change) and (ii) microstructural changes and clinical impairment (9HPT, ARAT, MVC) over and above any differences that could be attributed to cord area [1]. SPSS (SPSS Inc., Chicago, IL) was used for the multiple linear regression analysis and differences between mean FA of the ROIs between SCI subjects and controls. Results that survived p<0.05 are reported.

### Brain DTI Data

In a previous study, we reported the main effect of DTI metrics in the cranial CST that included this subset of participants [19]. Here, we present a further analysis, in which we tested whether spinal FA of the CST was associated with cranial FA of the CST. We constructed a General Linear Model (GLM), comprising the FA of the cervical CST as well as group effect and age and total intra-cranial volume confounds. The GLM was fitted to the registered and smoothed subject specific FA values and the resulting parameters were used to calculate a t*-*statistic at each voxel within the central CST. The t-tests were one-tailed and associated *p*-values were corrected for multiple comparisons within each ROI (Family Wise Error (FWE) *p*<0.05) using Gaussian random field theory [30].

The resulting statistical parametric maps (SPMs) allowed us to test for the main effect of spinal FA on cranial FA of the CST [19] and the interaction between spinal FA and trauma. The interaction was used to identify voxels in the cranial CST where trauma-related changes in FA could be explained by spinal FA, relative to normal variability.

## Results

### Clinical Data and Cord Area

Nine chronic SCI subjects (mean period post SCI was 14.8 years (SD 7.2, range 7–30) had lesions of the cervical cord (C5–C8) due to a traumatic event (eight fractures and one disc prolapse). All patients experienced bilateral impairment of the upper and lower limbs as assessed by the American Spinal Cord Injury Association Impairment classification (Tabel 1). Specifically, SCI participants had lower grip strength [SCI: mean = 0.13 mV (SD 0.1) vs. Controls: 0.49 mV (SD 0.26), *p* = 0.001] and performed more slowly on the 9HPT with the dominant hand [SCI: mean = 98.28 sec (SD 84.93) vs. Controls: mean = 17.04 sec (SD 1.56), p = 0.007] and non-dominant hand [SCI: mean = 141.22 sec (SD 121.98) vs. Controls: mean = 18.14 sec (SD 1.35), p = 0.005] when compared to controls (Table 1).

**Table 1:** Individual clinical and behavioural data for the SCI subjects with means.

Subject	Age	Aetiology of the injury	Time since injury (years)	Level of motor impairment/ASIA	dh 9HPT	ndh 9HPT	MVC	ARAT
1	43	fracture	14	C6/D	68.0	54.35	0.22	36.0
2	29	fracture	9	C6/B	52.6	59.2	0.05	42.0
3	44	fracture	7	C7/C	56.75	118.4	0.25	57.0
4	35	fracture	14	C5/A	190.5	300.0	0.02	26.0
5	61	fracture	19	C6/A	68.3	76.5	0.05	26.5
6	40	disc prolapse	19	C5/C	283	300.0	0.01	26.0
7	53	fracture	7	C8/D	38.55	42.45	0.25	53.0
8	56	fracture	15	C5/D	105.0	300.0	0.11	25.0
9	50	fracture	30	C5/D	21.8	20.1	0.22	57.0
Mean	45.7		14.9		98.3	141.2	0.13	38.72
SD	9.7		6.8		84.94	121.98	0.1	13.13

ASIA =  American Spinal Injury Association impairment scale; dh = dominant hand; ndh =  non-dominant hand; 9HPT =  Nine Hole Peg Test; MVC =  maximum voluntary contraction, ARAT =  Arm Research Arm Test; SD =  standard deviation.

As reported previously, the cross-sectional spinal cord area was decreased by more than 30% [1].

### Differences in FA between SCI Subjects and Controls

**Figure 2:**
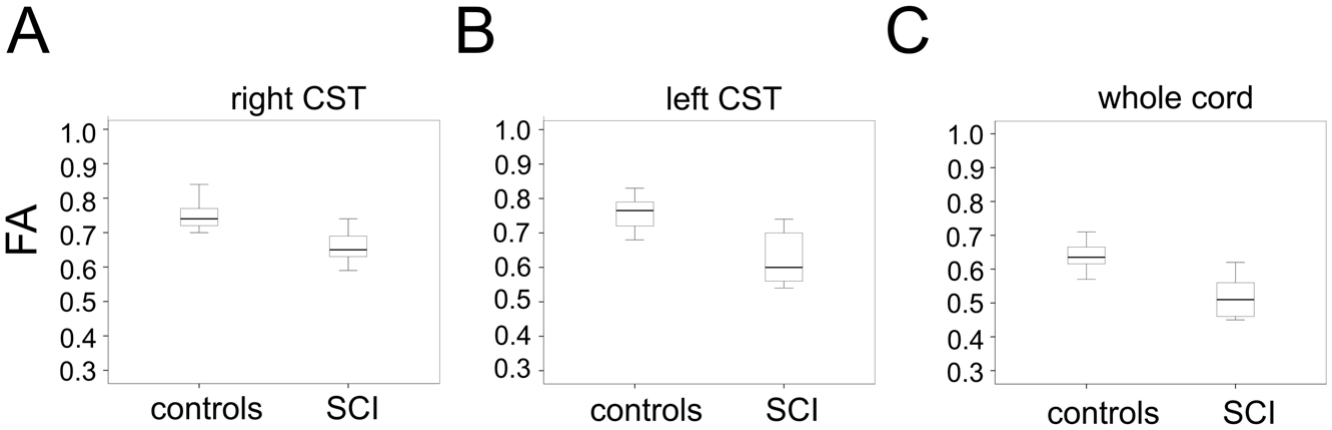
Box plots showing the statistically different mean FA in the region of interest in (A) the right corticospinal tract, (B) left corticospinal tract and (C) whole spinal cord area in controls and SCI patients.

Compared to controls, SCI subjects showed lower mean FA in right CST ROI [SCI: 0.67 (SD 0.07) vs. Controls: 0.75 (SD 0.04), p = 0.008] (Fig. 2 A), left CST ROI [SCI: 0.63 (SD 0.08) vs. Controls: 0.76 (SD 0.05), p<0.001] (Fig. 2B) and in the whole cervical cord ROI [SCI: 0.52 (SD 0.06) vs. Controls: 0.63 (SD 0.04), p<0.001] (Fig. 2 C).

Associations between cervical FA and

#### (i) cervical atrophy

**Figure 3:**
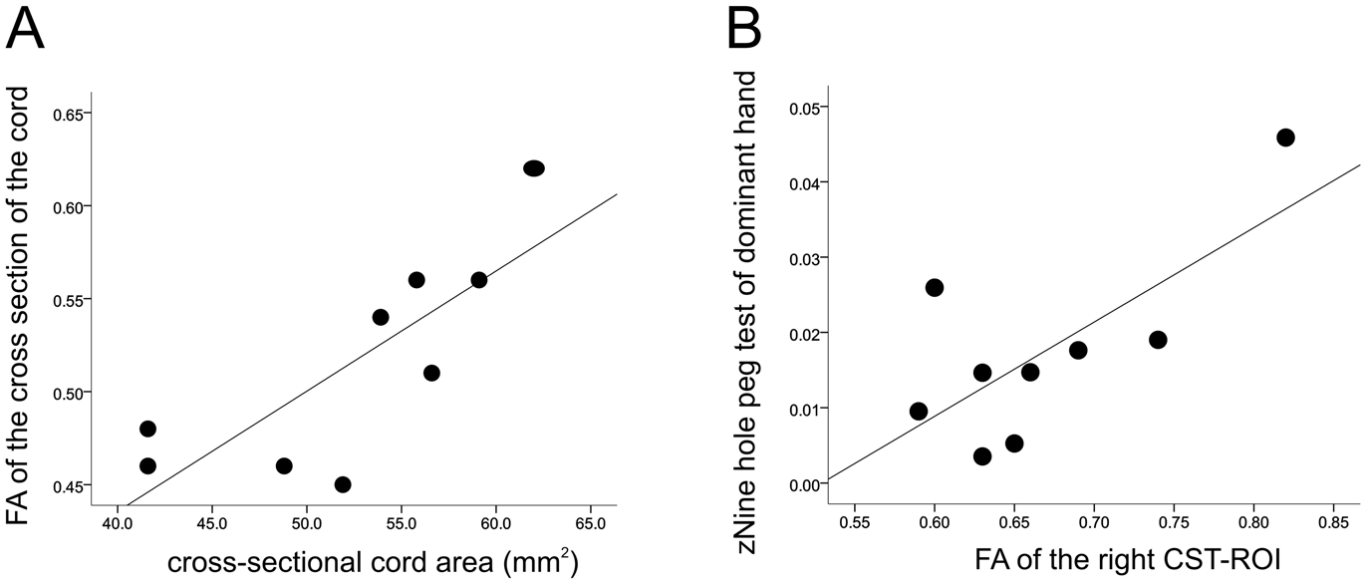
Scatter plots showing the correlations between spinal FA of the corticospinal tract and cord area and clinical measures in SCI subjects. (A) FA of the whole cervical cord ROI vs. cross sectional spinal cord area, (B) Spinal FA of the right CST-ROI vs. dominant 9HPT score. Note that greater values of the 9HPT score represent better outcome.

In SCI patients, the FA of the whole cervical cord ROI was positively correlated with cross-sectional spinal cord area (i.e. cord atrophy) (r = 0.80 p = 0.01), independently of age (Fig. 3 A).

#### (ii) upper limb function

Similarly, FA of the right CST ROI was positively correlated with the right 9HPT score independently of cross-sectional cord area and age (r = 0.72, p = 0.03) (Fig. 3 B). In other words, changes in spinal microstructure – reflected by FA – is associated with the degree of manual dexterity impairment independently of spinal cord atrophy and age.

#### (iii) FA of cranial CST

**Figure 4:**
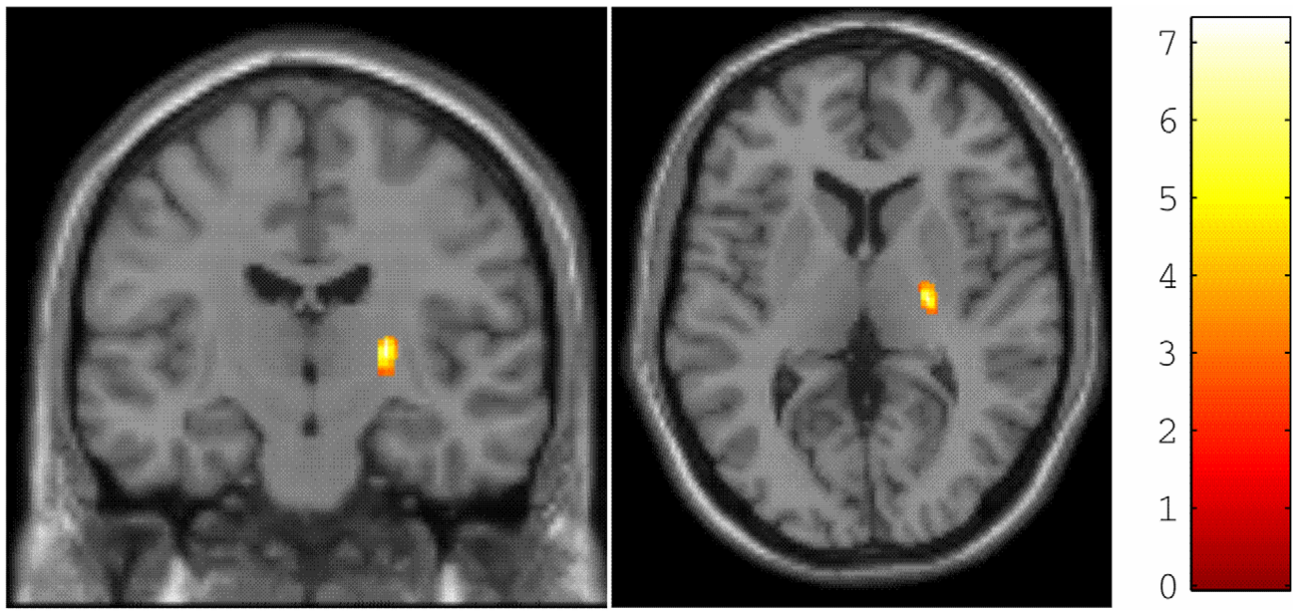
Statistical parametric maps (thresholded at p<0.01, uncorrected for display purposes only) showing the right internal capsule, in which changes in cranial FA of the corticospinal tract (CST) are more sensitive to spinal changes in FA of the mean CST compared with normal variability. The colour bar represents the t-value.

We first confirmed a decrease in FA at multiple levels of the central CST in SCI subjects compared to controls (see [19]) – comprising the bilateral pyramids and the posterior knee of the internal capsule. Crucially, here we found a significant interaction (x = 24, y = −15, z = 6; p = 0.001, FWE corrected) between the main effect of group and spinal FA (Fig. 4). In other words, changes in spinal FA of the CST following trauma were associated with greater changes in the FA of the posterior knee of the internal capsule, relative to changes under normal inter-subject variability.

## Discussion

This study establishes relationships between disruption of CST white matter – at a microstructural level, as demonstrated by fractional anisotropy, and spinal cord area – and disability. In addition we observed reductions of FA in the central nervous system, representing remote correlates of degeneration in the chronically injured cervical cord.

### Axonal Degeneration in the Injured Cervical Cord

It is now well established that trauma to the spinal cord results in macrostructural changes leading to spinal and cortical atrophy [1], [2], [31]. The pathological processes underlying these changes are not fully resolved and may be the result of multiple microstructural changes such as axonal degeneration [7], progressive demyelination [32], loss of large diameter axons [33] death of oligodendrocytes [34] and/or damage to the spinal grey matter [35]. We found lower FA in the CST at cervical segments rostral to the traumatic impact and also a general reduction of FA in the cross-section of the cervical cord including white and grey matter, in agreement with previous reports [3], [36]. These alterations of the axonal architecture suggest that multiple processes, such as axonal degeneration and demyelination, might occur at sites several segments rostral to trauma to the cervical cord.

### (i) *Association* between Lower FA and Spinal Atrophy

Moving beyond the group differences, our aim was to determine the relationship between micro– and macrostructural effects of a traumatic SCI. Reduced cord area (i.e. spinal atrophy) predicts upper limb impairment following SCI [1], [6] and relates to white matter changes of the cranial CST [19]. Here, we add to this finding by reporting a relationship between FA in the cervical cord and cord area at the identical anatomical level. The link between micro- and macrostructure may be helpful in understanding the time course of changes at different structural levels. In particularly, acute changes in FA may precede a decline in cord area [1], [6] as the latter results from an accumulation of microstructural as well as macrostructural changes representing the endpoint of neurodegeneration. Future studies may investigate the rate of the underlying changes – that may help understand the underlying pathophysiology.

### (ii) *Association* between Axonal Degeneration and Upper Limb Function

Recent studies investigating structural spinal changes have demonstrated a close relationship between cervical microstructural changes and the ASIA motor scale [3], [37]. The ASIA score quantifies gross motor and sensory changes – while it is anticipated that treatment strategies will induce only minimal functional changes [38]. Therefore, we assessed fine finger movement and showed that lower mean FA measured in ROIs that correspond to the left and right lateral CST predicts impaired manual dexterity – as assessed by the performance during the 9HPT – independently of cord atrophy [1]. This means that a reduced FA, reflecting microstructural tissue changes within ROIs of the CST, may be a complementary predictor of fine motor recovery, in addition to cross-sectional spinal cord area measurement.

### (iii) *Association* between Changes in Microstructure of the CST at Spinal and Cranial Level

Previously, we found – in the same patient cohort – a significant relationship between macrostructural spinal changes (i.e. atrophy) and microstructural changes of the cranial CST [19]. Here, we provide a further mechanistic insight by demonstrating a relationship between axonal degeneration in the CST, a tract indispensable for voluntary control of manual dexterity [39], immediately rostral to the initial site of trauma and changes upstream in the CST at the level of the internal capsule. Based on animal literature [39], trauma induces retrograde axonal degeneration that, over time, leads to quantifiable changes in white matter tissue at multiple levels of the CST. Moreover, the association between reduced micro- and macrostructural integrity in the spinal cord and brain [19] supports our recent finding, in the same patient cohort, of changes of the electrophysiological properties of the CST reflected by increased motor threshold and prolonged cortical silent periods [5]. In other words, trauma induced retrograde axonal degeneration of the CST – as detected by DTI – may be a direct correlate of impaired corticospinal excitability, as reflected by increased motor thresholds [5], [37].

Finally, it should be noted that measures of neural changes of the spinal cord and brain at the microstructural level can be influenced, in addition to trauma, by different degrees of activity, such as suppression (i.e. immobilization) or enhancement (i.e. training) throughout the patients clinical history. For example, in patients with immobilization of the hand (in writer’s cramp) or upper limb (in cast treatment due to fracture) a relative decrease in grey matter occurred in the contralateral M1 along with a decrease in corticomotor excitability [40]. Subsequent training reversed the effects of immobilization, and resulted in a re-establishment in regional grey matter density and excitability of M1. Similar activity-dependent effects over time could have influenced, structural changes in these chronic SCI patients. Future studies will assess the influence of intensity (time spent in training, frequency, duration, repetitions, etc.) and nature of rehabilitation interventions (active versus passive movements, task orientation etc) as well as the overall levels of activity (post-traumatic immobilization versus mobilization into wheelchair or locomotion).

In conclusion, we have demonstrated that trauma induces a reduction in structural integrity in the CST throughout the cervical cord and brain. Crucially, the amount of degeneration is associated with clinical status in chronic SCI, independently of spinal atrophy. Thus, our clinically viable MRI protocol allows the assessment of long-distance fibre degeneration. Future longitudinal studies, in larger cohorts of SCI subjects, are necessary to investigate whether DTI metrics can serve as sensitive biomarkers but the results of this study – involving only a small group – provide promising initial results.
